# Hearing Loss as a Function of Aging and Diabetes Mellitus: A Cross Sectional Study

**DOI:** 10.1371/journal.pone.0116161

**Published:** 2014-12-30

**Authors:** In-Hwan Oh, Jong Hoon Lee, Dong Choon Park, MyungGu Kim, Ji Hyun Chung, Sang Hoon Kim, Seung Geun Yeo

**Affiliations:** 1 Department of Preventive Medicine, School of Medicine, Kyung Hee University, Seoul, Korea; 2 Department of Radiation Oncology, St. Vincent’s Hospital, College of Medicine, The Catholic University of Korea, Suwon, Korea; 3 Department of Obstetrics and Gynecology, St. Vincent’s Hospital, College of Medicine, The Catholic University of Korea, Suwon, Korea; 4 Department of Otorhinolaryngology-Head and Neck Surgery, Samsung Changwon Hospital, Sungkyunkwan University School of Medicine, Changwon, Korea; 5 Department of Otorhinolaryngology-Head and Neck Surgery, School of Medicine, Kyung Hee University, Seoul, Korea; Sun Yat-sen University, China

## Abstract

**Background:**

Although hearing loss may be caused by various factors, it is also a natural phenomenon associated with the aging process. This study was designed to assess the contributions of diabetes mellitus (DM) and hypertension, both chronic diseases associated with aging, as well as aging itself, to hearing loss in health screening examinees.

**Methods:**

This study included 37,773 individuals who underwent health screening examinations from 2009 to 2012. The relationships between hearing threshold and subject age, hearing threshold at each frequency based on age group, the degree of hearing loss and the presence or absence of hypertension and DM were evaluated.

**Results:**

The prevalence of hearing loss increased with age, being 1.6%, 1.8%, 4.6%, 14.0%, 30.8%, and 49.2% in subjects in their twenties, thirties, forties, fifties, sixties, and seventies, respectively (p<0.05). Hearing value per frequency showed aging-based changes, in the order of 6000, 4000, 2000, 1000 and 500 Hz, indicating greater hearing losses at high frequencies. The degree of hearing loss ranged from mild to severe. Aging and DM were correlated with the prevalence of hearing loss (p<0.05). There was no statistically significant association between hearing loss and hypertension after adjusting for age and DM.

**Conclusions:**

The prevalence of hearing loss increases with age and the presence of DM. Hearing loss was greatest at high frequencies. In all age groups, mild hearing loss was the most common form of hearing loss.

## Introduction

Aging refers to the accumulation of changes that occur from the moment of fertilization to the death of an organism, and is a universal and nonreciprocal process over time. Although the speed of aging differs in different individuals and in different organs of an individual, the aging process is inevitable.

Hearing organs experience aging-associated degenerative changes, with aging being the most frequent cause of sensorineural hearing loss in adults. Hearing loss is regarded not only as a communication disorder, but as a major disease that severely impairs patient quality of life, due to social withdrawal, psychological alienation, loss of confidence, and increased depression and anxiety [1]. Age related hearing loss is one of the three leading common chronic diseases in elderly individuals, along with arthritis and hypertension, and its incidence is increasing rapidly. More than 35% of people in their sixties and 50% of those in their seventies have difficulties in daily activities due to hearing loss [2]. Age-related hearing loss is generally due to aging-related changes in hearing apparatus, although other causes have been identified, including noise, chronic diseases, such as hypertension, hyperlipidemia, diabetes mellitus (DM) and renal disease, as well as hereditary factors. To date, however, the exact causes and mechanisms of hearing loss have not been clarified [3].

Degenerative diseases, including coronary artery disease, DM, hypertension, cancer, senile dementia, arthritis, and osteoporosis, interact with the aging process. Thus, good lifestyle habits can delay the appearance of chronic diseases, resulting in a better quality of life with shorter periods of sickness even with increasing life expectancy [4]. Aging of the hearing apparatus may also interact with other degenerative diseases, with various studies attempting to assess these correlations.

Although many studies have attempted to assess the correlations of DM and hypertension with hearing loss, these correlations remain unclear. Body mass index (BMI), however, has been reported to correlate with hearing loss, but there have been difficulties determining whether hearing loss correlates with BMI-associated DM and hypertension since increased BMI accompanies DM and hypertension in many patients. To better understand these relationships, this study assessed the prevalence of age-related hearing loss and whether hearing loss was associated with DM and hypertension, in a group of normal and overweight (BMI 18.5∼25 kg/m^2^) individuals, using findings on hearing tests performed at a health promotion center.

## Methods

### Subjects

This cross sectional study involved 37,773 individuals who visited the Health Promotion Center for health screening examinations from 2006 to 2012. The subjects included 28,672 males and 9,101 females, of mean age 44.1±8.4 years. Individuals with asymmetric hearing loss, trauma, auricular anomaly, head and neck deformity, otitis media or a history of ear surgery, congenital or childhood hearing loss were excluded. This study is a case-control study using medical data. Health records were anonymized prior to use. The institutional review board waived the consent procedure because of the retrospective nature of the study. This study was approved by the Institutional Review Board of Kyung Hee University (IRB No. KMC IRB 1326-02).

### Hearing Test

Hearing thresholds were examined at frequencies of 500, 1,000, 2,000, 3,000, 4,000 and 6,000 Hz using an Auricle Plus audiometer (GN Otometrics, Copenhagen, Denmark) in a double-walled sound booth, in accordance with the guidelines for pure tone averages (PTA) of the Korea Occupational Safety and Health Agency (KOSHA).

Frequencies were measured starting at 1,000 Hz and in the order of 2,000 Hz, 3,000 Hz, 4,000 Hz and 6,000 Hz. The subjects were re-examined at a frequency of 1,000 Hz and air conduction thresholds were measured at a frequency of 500 Hz and then at 250 Hz.

Degrees of hearing were based on the standards for noise-induced hearing loss and communication disorders. Six-dimensional methods of PTAs were determined by measuring the sum of the air conduction thresholds of the right and left ears at four frequencies, 500, 1,000, 2,000 and 4,000 Hz, frequencies frequently measured clinically and in the industrial sector and calculated as (500 Hz+2×1,000 Hz+2×2,000 Hz+4,000 Hz)/6.

The average of both ears was used for analysis. The PTA of both ears at each frequency was obtained for frequency analysis. An average hearing threshold ≥26 dB was defined as hearing loss and the degree of hearing loss was categorized as mild (26∼40 dB), moderate (41∼55 dB), moderately severe (56∼70 dB) and severe (71∼90 dB) based on the standard of ISO. The average hearing threshold in each age group and the hearing threshold at each frequency were also determined [5].

### Blood Pressure Test

Blood pressure was measured using a standardized mercury manometer after subjects had rested for 20 minutes on an empty stomach.

Blood pressure was measured with an automatic blood pressure monitor; if abnormal, it was again measured manually. Based on the WHO Criteria (1978), hypertension was defined as a systolic blood pressure ≥140 mmHg or a diastolic blood pressure ≥90 mmHg.

### Blood Chemistry Tests

Subjects were asked to avoid vigorous exercise or alcohol consumption for 24 hours, and to maintain a fasting state for at least 8 hours prior to blood sample collection. Blood samples were collected into anticoagulant-free tubes (BD vacutainer, Korea) and analyzed with an automatic hematology analyzer (Modular DP, Roche Diagnostics, Switzerland).

DM was defined according to American Diabetes Association criteria as a normal fasting blood glucose concentration ≥126 mg/dl or an HbA1c level ≥6.5%.

### Statistical analysis

The prevalence and severity of hearing loss were analyzed in each group of subjects. Subjects with hearing loss were also subclassified by its severity (mild, moderate, moderately severe or severe) and the relationship between risk factors and hearing loss in each age group was determined. As the association between hearing loss and BMI independent of DM is unclear, analysis was limited to subjects with normal BMI.

To assess the risk factors associated with hearing loss, subjects were divided into two groups: those with normal hearing and those with hearing loss. Between group differences in age and prevalence rates of hypertension and DM were compared using t-tests and Chi-square tests. Univariate and multivariate logistic regression analyses were performed to determine the crude and adjusted odds ratios (ORs) of age, hypertension, DM and gender as factors associated with hearing loss. All statistical analyses were performed using IBM SPSS version 20 (IBM Corp., Armonk, NY), with a p value <0.05 defined as statistically significant.

## Results

**Figure 1:**
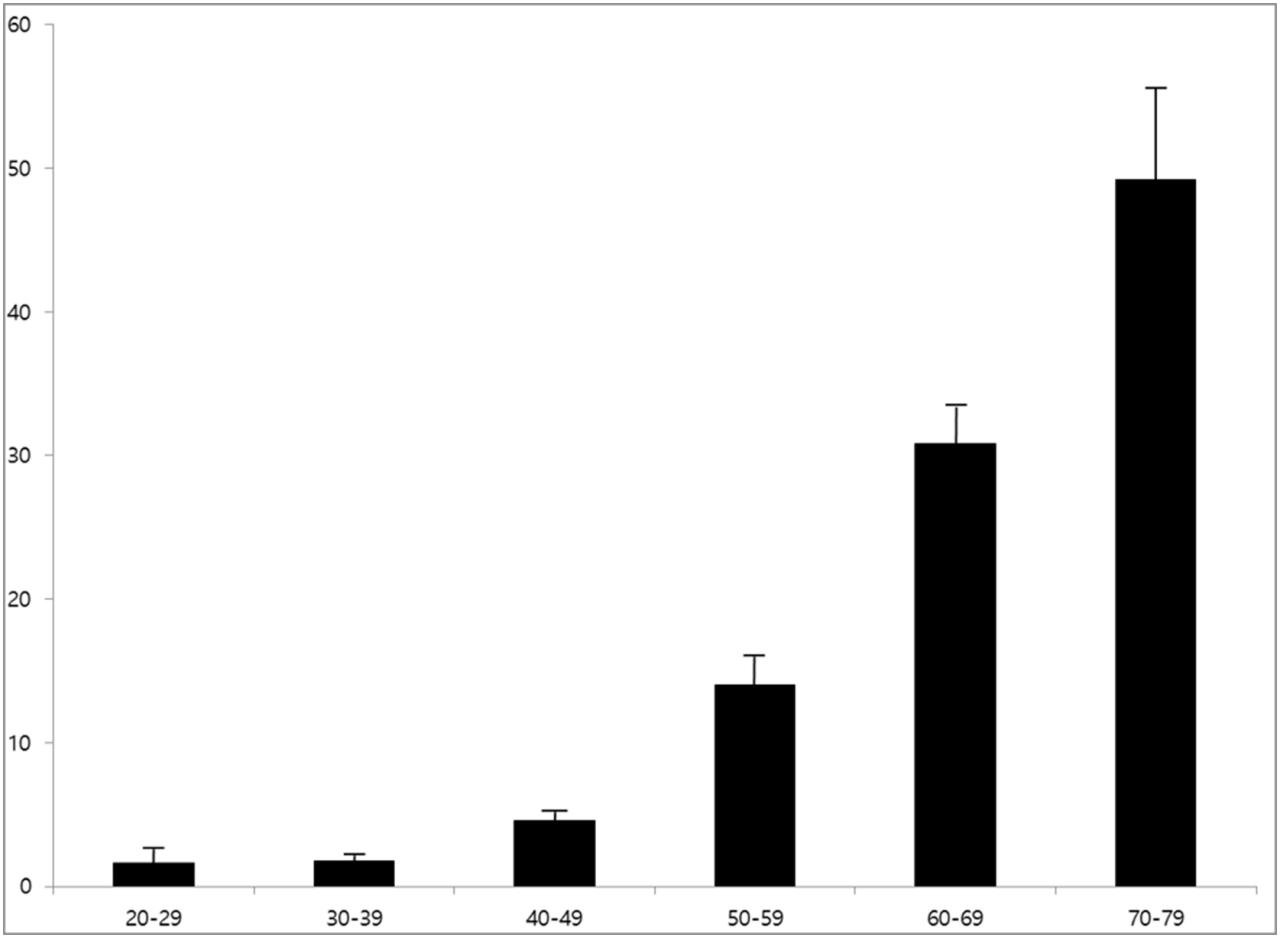
Prevalence of hearing loss by age group.

The prevalence rates of hearing loss significantly increased with age, being 1.6%, 1.8%, 4.6%, 14.0%, 30.8%, and 49.2% in subjects in their twenties, thirties, forties, fifties, sixties and seventies, respectively (p<0.05) (Fig. 1; Table 1). Defining a hearing threshold <26 dB as normal, abnormal findings were found at 6 kHz for subjects in their twenties and thirties, at 4–6 kHz for subjects in their forties and fifties, at 2–6 kHz for subjects in their sixties and at 0.5–6 kHz for subjects in their seventies. Hearing thresholds showed greater increases at high frequencies, indicating that age-associated hearing loss progresses relatively earlier in high frequency regions (Table 1).

**Table 1:** Pure tone thresholds at each frequency stratified by age.

Age		500 Hz (Lt)	500 Hz (Rt)	1000 Hz (Lt)	1000 Hz (Rt)	2000 Hz (Lt)	2000 Hz (Rt)	4000 Hz (Lt)	4000 H z (Rt)	6000 Hz (Lt)	6000 Hz (Rt)
20s (n = 315)	Average	10.84	10.24	9.48	8.76	10.08	9.48	12.97	12.43	27.10	24.78
	SD	7.17	6.95	8.34	6.45	10.06	9.27	13.26	13.22	17.90	16.54
30s (n = 11397)	Average	11.94	12.22	10.61	10.80	11.32	11.26	19.71	19.03	31.23	30.34
	SD	8.81	9.31	8.70	8.97	9.47	9.51	17.48	17.62	18.48	18.24
40s (n = 16604)	Average	13.65	13.95	13.08	13.21	14.75	14.34	28.10	27.22	37.53	37.00
	SD	10.56	11.00	10.73	10.96	11.79	11.92	19.87	20.29	18.83	19.26
50s (n = 7858)	Average	16.37	16.57	16.84	16.82	20.33	19.50	37.67	35.86	44.39	43.23
	SD	12.84	12.47	13.57	12.86	15.54	14.77	21.18	21.15	19.41	19.59
60s (n = 1229)	Average	21.21	21.73	21.44	22.23	26.77	26.54	43.65	41.82	52.54	52.35
	SD	15.31	15.25	15.40	15.56	17.35	16.72	20.62	21.33	19.21	19.05
70s (n = 364)	Average	26.72	28.63	27.39	29.23	33.61	34.55	48.45	48.34	58.32	59.51
	SD	17.39	18.62	17.14	18.09	17.29	17.40	18.11	20.21	18.38	19.62
Total (n = 37773)	Average	14.05	14.33	13.50	13.64	15.41	15.03	28.13	27.09	37.66	36.90
	SD	11.11	11.37	11.48	11.51	13.03	12.84	20.78	20.90	19.73	19.91

N; number, SD; standard deviation, Hz; Hertz, Rt; Right, Lt; Left.

**Figure 2:**
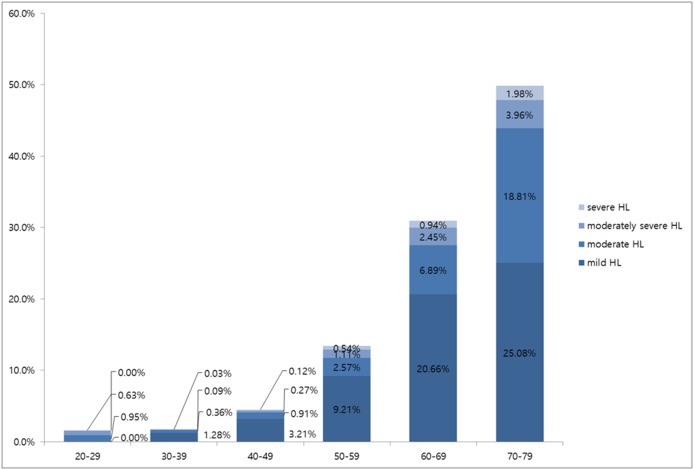
Prevalence of hearing loss according to the severity of hearing loss by age group in subjects without DM.

**Figure 3:**
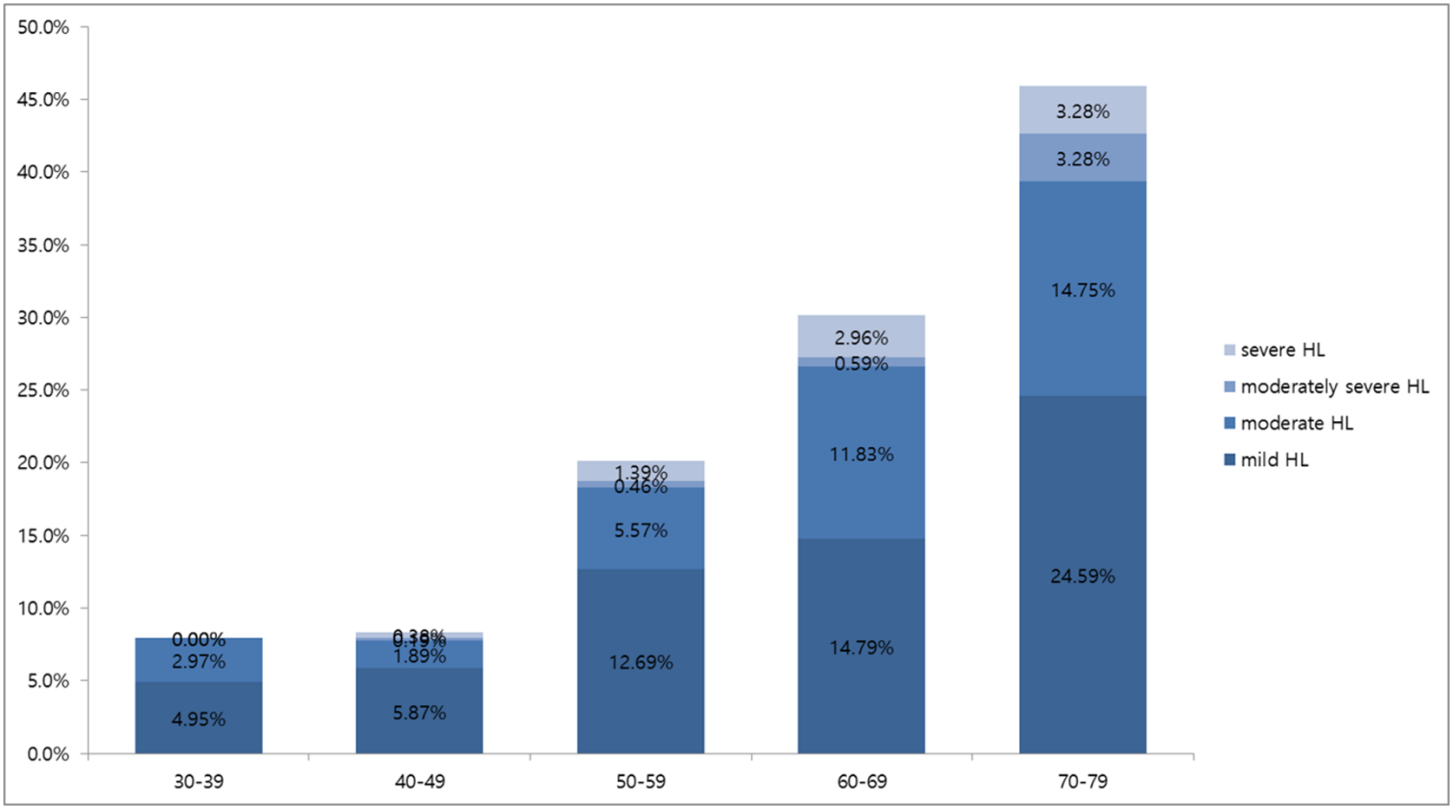
Prevalence of hearing loss according to the severity of hearing loss by age group in patients with DM.

Mild, moderate, moderately severe and severe hearing loss was observed in 0.0%, 0.9%, 0.6%, and 0.0%, respectively, of subjects in their twenties; in 1.3%, 0.4%, 0.1%, and 0.03%, respectively, of subjects in their thirties; in 3.3%, 0.9%, 0.3%, and 0.1%, respectively, of subjects in their forties; in 9.5%, 2.8%, 1.1%, and 0.5%, respectively, of subjects in their fifties; in 19.9%, 7.6%, 2.2%, and 1.2%, respectively, of subjects in their sixties; and in 25.0%, 18.1%, 3.8%, and 2.2%, respectively, of subjects in their seventies (Fig. 2), showing an age-related increase in severity of hearing loss (p<0.05). In all age groups, the degree of hearing loss showed the same sequential frequency, being mild, moderate, moderately severe and severe, in that order. Fig. 3 shows the prevalence of hearing loss among DM patients. In all age groups, mild hearing loss was the most common form of hearing loss (Fig. 3).

Mean age was greater in subjects with hearing loss than in the normal hearing group (52.8±9.8 years vs. 43.45±7.98 years, p<0.001).

The prevalence rates of hearing loss were significantly greater in subjects with than without hypertension (10.9% vs. 6.6%, p<0.05) and in subjects with than without DM (17.3% vs. 6.5%, p<0.05) (Table 2).

**Table 2:** Baseline characteristics of hearing disturbance and normal groups.

Risk factors		Normal hearing		Hearing disturbance		p-value*
		N = 35137 (93.02%)		N = 2636 (6.98%)		
		Average/n	SD/%	Average/n	SD/%	
Age		43.45	7.98	52.81	9.85	<.0001
HTN	(−)	31896	93.4	2239	6.6	<.0001
	(+)	3241	89.1	397	10.9	
DM	(−)	33893	93.5	2375	6.5	<.0001
	(+)	1244	82.7	261	17.3	

HTN; Hypertension, DM; Diabetes Mellitus,

*T-test& Chi-square test were used.

Univariate analysis showed that age, DM and hypertension were significantly associated with hearing loss (p<0.05). Multivariate analysis showed that DM was an independent predictor of hearing loss (p<0.05), whereas hypertension was not (p>0.05; Table 3).

**Table 3:** Crude and adjusted odds ratios of hearing disturbance among Korean adults.

	Univariate analysis		Multivariate analysis^a^	
	Odds Ratio	Confidence Interval	Odds Ratio	Confidence Interval
Age	1.121*	1.116–1.126	1.119*	1.114–1.125
HTN	1.745*	1.559–1.953	1.098	0.934–1.830
DM	2.994*	2.604–3.443	1.398*	1.200–1.628

*statistically significant, HTN: hypertension, DM: diabetes mellitus.

a Including the following variables: age, sex, HTN, DM.

## Discussion

The aging of hearing organs is due to degenerative changes, resulting in hearing loss. Its age of onset and degree of progression are determined by both genetic and environmental factors. Aging of the hearing apparatus is characterized by bilateral symmetric sensorineural hearing loss in high frequency regions, ≥2 kHz. Moreover, males are more severely affected than females [6]. Daily activities are not interrupted during early stages of hearing loss, since the aged organs are not impaired at daily speech ranges of 500∼2,000 Hz. In high frequency regions, however, distinguishing consonant sounds and speech discrimination gradually become more difficult, leading to difficulties in sound discrimination, especially in crowded and noisy places.

In the United States, 80% of individuals with hearing loss are aged ≥65 years, with these subjects having at least a four-fold higher incidence of hearing loss than other age groups. Moreover, some degree of hearing loss is observed in almost all subjects aged >70 years [7]. The prevalence of age-related hearing loss varies by ethnicity, as well as by age, level of hearing loss, and criteria of hearing loss. For example, in Korea, 45.8% and 14.8% of subjects aged >64 years had hearing thresholds ≥26 dB and >40 dB, respectively [5]. In the United States, 29% of subjects, including 32.5% of males and 26.7% of females aged >62 years had hearing thresholds ≥26 dB; in the United Kingdom, 92% and 31% of people aged >59 years had hearing thresholds ≥25 dB and ≥45 dB; and in Taiwan, 96.5%, 38.7% and 27.2% of people aged >64 years had hearing thresholds ≥25 dB, ≥40 dB, and ≥55 dB, respectively [5].

In this study, normal hearing was defined as a 0–25 dB range and hearing loss as ≥26 dB. Using these criteria, the prevalence of hearing loss increased with age, ranging from 1.6% for subjects in their twenties to 49.2% for subjects in their seventies. The most frequent degree of hearing loss was mild, although all levels of hearing loss increased with age. Abnormal findings at 6 kHz were observed in all age groups; as age increased, abnormal findings were observed in the order 4 kHz, 2 kHz, 1 kHz and 500 Hz. These findings indicate that hearing loss starts at high frequency, occurring later at the frequency of normal human speech and then at low frequency.

Sound is perceived through a complicated pathway, and functional changes in the composition of this pathway weaken hearing ability with age. Although aging-associated hearing loss is thought to be associated with the aging of auditory hair cells and neurons, and the degeneration of angioid streaks, the exact mechanism of degeneration remains unclear. About 15% of elderly people have motility disorders, regardless of the presence or absence of neurologic disease. Hair cell defects, tissue degeneration, and apoptosis in the cochlear lateral wall have been detected histologically in the ears of elderly subjects. These anatomical changes in auditory organs may be caused by physiological changes that affect hearing function [8]. Such aging of the hearing apparatus is also interconnected with environmental and genetic factors [14].

Aging-associated hearing loss is due primarily to genetic factors, but other factors may be involved, including exposure to noise, ear infections, smoking, hormones, gender, ototoxic substances, head trauma, cardiovascular disease (hypertension, atherosclerosis and hyperlipidemia), plasma hyperviscosity, DM, immune function impairment, metabolic bone disease, renal failure, endocrine medical conditions, Alzheimer’s disease, bone mineral density, ontological conditions such as Meniere’s disease and otosclerosis and as yet unidentified causes [1], [9]. Of these causes, DM and hypertension, which are common degenerative diseases frequently accompanying aging, have been reported to be closely related to aging-related hearing loss, although other studies have reported no such correlations [10], [11].

Previous studies assessing the correlation between hypertension and hearing loss differ by study subjects, study methods, and analytic methods. Most studies showed a correlation between hearing loss and aging-related hypertension but one study, in the Sudanese native population, reported no correlation [12], whereas another study reported that the incidence of hearing loss in hypertensive patients of mean age >75 years was not high [13]. Differences among study outcomes may be the result of factors that can affect hearing loss, including noise exposure; inhalation of toxic substances; ingestion of ototoxic drugs and metabolic and circulatory substances; infections, injuries; genetic factors; chronic diseases; lifestyle and aging itself [14], [15]. Although hypertension has been reported to be unrelated to hearing loss, three factors suggest that such an association should exist. First, high pressure in the vascular system could cause hemorrhaging in the cochlear and anterior vestibular arteries, both present in the inner ear and branching from the anterior inferior cerebellar artery. Second, increased blood viscosity could induce tissue hypoxia by decreasing capillary blood flow and oxygen transport. Third, arterial hypertension could cause progressive or sudden hearing loss by inducing ionic changes in cell potentials [16]–[18]. In this study of subjects who underwent health screening examinations, the prevalence of hearing loss was significantly higher in those with than without hypertension (10.9% vs. 6.6%), although multivariate analysis showed that hypertension was not an independent predictor of hearing loss.

The incidence of DM, a chronic disease in adults, is increasing worldwide. DM has been reported to induce progressive bilateral sensorineural hearing loss with aspects similar to presbycusis, including greater hearing loss at high frequencies. DM is frequently accompanied by complications, including neuropathy, retinopathy, nephropathy, ischemic heart disease and hypertension [19]. Therefore, hearing loss is more likely to occur in patients with than without DM and to worsen in subjects with uncontrolled DM and in patients with complications. Most studies have reported a correlation between DM and hearing loss. Studies that observed no correlation have found no differences in hearing threshold between DM patients and age-matched comparison groups [10], [11]. Those studies, however, did not assess the duration of DM, blood sugar levels, age and the degree of DM management as factors associated with hearing loss.

Studies reporting no correlation between DM and hearing threshold have found that hearing threshold at low frequency was correlated with serum glucose concentration, with hearing thresholds increasing with increasing serum glucose [20]. Moreover, a study that excluded subjects with aging-related hearing loss found a significant correlation between DM and hearing threshold [21].

Many studies have reported a close correlation between DM and hearing loss, with DM patients showing a higher incidence of sensorineural hearing loss. These studies include a study in which hearing was normal during early stages of DM, whereas 30% of patients with chronic DM had hearing loss [22], a study showing that hearing loss due to peripheral nerve injury was greater in patients with type 2 than type 1 DM [23]; a study in which patients with DM-related complications showed significant delays in both the absolute and interpeak latency of wave I-V and decreased amplitude on auditory brainstem response (ABR) tests [24]; a study in which not only the ABR but the DPOAE of the DM group showed abnormal findings [25]; a study in which abnormal findings were found on pure tone audiometry and ABR tests [26]; a study in which elderly subjects with DM showed greater hearing loss at low frequency and hearing loss in patients with DM rapidly worsens with aging [27]; and a study in which veterans under the age of 60 years with DM showed significantly higher incidence of hearing loss for high frequency from noise [28]. As there is a correlation between hearing loss and DM, more attention should be paid to DM in the counselling and rehabilitation of hearing loss patients.

We found that the prevalence of hearing loss was significantly higher in subjects with than without DM (17.3% vs. 6.5%, p<0.05). In addition, multivariate analysis showed that DM was a significant predictor of hearing loss (OR 1.398). The potential mechanisms of hearing loss from DM include damage to the cochlea from circulatory disorders of the inner ear; retrocochlear hearing loss caused by neuritis of the auditory nerve; the occurrence of diabetic neuropathy; and mitochondrial DNA mutations [29]. Not all patients with DM, however, experience hearing loss, and treatment of DM (e.g., by administration of hypoglycemic agents, regular exercise and better eating habits) may reduce the incidence of hearing loss in some patients with DM.

This study had several limitations. First, due to its cross sectional nature, this study assessed subjects born at different times, with different nutritional states, environments, and social, economic, and cultural backgrounds. Second, limiting subjects to health screen examinees may have introduced a selection bias. Third, various factors associated with aging were not considered, including smoking, exercise, drinking, drug use, chronic diseases, noise exposure and occupation.

## Conclusion

This study showed that the prevalence of hearing loss increases with age, as well as being higher in subjects with than without DM.
